# Circular RNA CEP128 acts as a sponge of miR-145-5p in promoting the bladder cancer progression via regulating *SOX11*

**DOI:** 10.1186/s10020-018-0039-0

**Published:** 2018-07-31

**Authors:** Zhun Wu, Wei Huang, Xuegang Wang, Tao Wang, Yuedong Chen, Bin Chen, Rongfu Liu, Peide Bai, Jinchun Xing

**Affiliations:** Department of Urology, the First Affiliated Hospital of Xiamen University, No.55 Zhenhai Road, Xiamen, 361003 Fujian China

**Keywords:** Bladder cancer, Circle RNA, circCEP128, miR-145-5p, *SOX11*

## Abstract

**Background:**

This study aimed to investigate the effect of over-expressing circular RNA CEP128 (circCEP128) on cell functions and explore the molecular mechanism of which in bladder carcinoma.

**Methods:**

The differentially expressed circRNAs and mRNAs in bladder carcinoma cells and cells in adjacent tissues were screened out using microarray analysis. Expression levels of circRNAs and mRNAs in tissues and cells were determined by qRT-PCR. Expression of *SOX11* was detected by western blot. Luciferase reporter assay and RNA pull-down assay were used to investigate the interactions between the specific circRNA, miRNA and mRNA. Cell cycle and apoptosis were measured using flow cytometry after transfection. MTT assay was also performed to detect the cell proliferation.

**Results:**

In present study, circCEP128 and *SOX11* were observed significantly up-regulated in bladder cancer tissues, while the expression of miR-145-5p was decreased in cancer samples compared to normal samples. Cytoscape was used to visualize circCEP128-miRNA-target gene interactions based on the TargetScan and circular RNA interactome, which revealed that circCEP128 served as a sponge of miR-145-5p and indirectly regulated *SOX11*. Knockdown of circCEP128 induced the inhibition of cell proliferation and the increased bladder cancer cell apoptosis rate.

**Conclusions:**

CircCEP128 functions as a ceRNA for miR-145-5p, which could up regulates *SOX11* and further promotes cell proliferation and inhibits cell apoptosis of bladder cancer.

## Background

Global Cancer Statistics alarms that bladder cancer, a tumor in urinary system, caused 130,000 deaths each year (Li et al., 2017). In China, the mortality and morbidity of bladder cancer ranked first among all the tumors in urinary system (Huang et al., 2016). Especially for the patients suffer from muscle-invasive bladder cancer, the occurrence of metastasis is more frequently and the prognosis is poorer than other kinds of bladder cancers (Zhong et al., 2017). Besides, most clinical trials of chemotherapy for advanced bladder carcinoma displayed limited benefits. Therefore, it is urgent for us to identify novel molecular targets for inhibiting the tumorigenesis of bladder cancer (Zhong et al., 2016).

MicroRNAs (miRNAs) are endogenous small noncoding RNA molecules (19–22 nucleotides in length) that negatively modulate protein-coding genes expression through binding to the specific sequence of genes (Enokida et al., 2016). Increasing evidences have demonstrated that miRNAs usually expressed abnormally in bladder cancer and resulted in multiple alterations during the development of cancer, such as promoting or inhibiting carcinogenesis (Li et al., 2017). MicroRNA-145 (miR-145) has been frequently reported to be down-regulated in various human cancers, including prostate cancer, bladder cancer and colon cancer, as well as in B-cell malignancies (Noguchi et al., 2013). Exogenous miR-145 has also been speculated as tumor inhibitor when being administrated intravesically (Minami et al., 2017).

Circular RNAs (circRNAs), without cap and polyA tails, forms covalently closed continuous loops by non-sequential back-splicing of pre-mRNA transcripts. They generally expressed in eukaryotic cells, but the biological function of them remains unclear (Zhong et al., 2017). CircRNAs are less prone to degrade since they are resistant to exonuclease, the property of which makes circRNAs superior than linear RNAs in terms of biological markers (Huang et al., 2016). Multiple properties of circRNAs have been identified in recent years, among which “miRNA sponges” role was most frequently discussed (Shang et al., 2016). Zhong et al., 2017 investigated competing endogenous RNA mechanism in bladder cancer, among which circRNA MYLK was profoundly discussed but circCEP128 was neglected (Zhong et al., 2017). CircTCF25 and circHIPK3 have been fully illustrated in other studies about ceRNA network and variant pathways (Li et al., 2017; Huang et al., 2016; Zhong et al., 2016). However, this speculation refers to cirCEP128 as a new bio-functional marker which has a close interaction with miR-145-5p in bladder cancer.

*SOX11*, as an intronless gene regulates cell fate, functions in tumorigenesis and adult neurogenesis. It is known to promote tissue remodeling, progenitor cell expansion and differentiation of a number of cell types, including neural progenitor cells (Oliemuller et al., 2017). *SOX11* is a diagnostic and prognostic antigen in B cell lymphomas and has recently been demonstrated to have tumor suppressor functions (Sernbo et al., 2011). It was found that miR-223-3p inhibitor restrains ovarian cancer development by increasing *SOX11* expression (Fang et al., 2017). Therefore, we hypothesized that *SOX11* might be regulated by miR-145-5p in human bladder cancer.

In present study, the expression profiles of circRNA and miRNA in cells of bladder tumor and adjacent tissues were illustrated clearly. The expression of SOX11 was also detected. The directly interactions among circRNA, miRNA and SOX11 were confirmed by luciferase assay. We hypothesized that circCEP128 might function as a competing endogenous RNA (ceRNA) for miR-145-5p in regulating *SOX11*, which might further inhibit cell proliferation and promote cell apoptosis of bladder cancer. The study might be of biological and clinical importance.

## Methods

### Clinical specimens

Ten pairs of bladder tumor tissues and adjacent bladder tissues were obtained from patients who suffered from radical cystectomy at the First Affiliated Hospital of Xiamen University. The normal bladder urothelium samples were collected with a distance of ≥3 cm from the edge of cancer tissues in the resected bladder. All the specimens for histological and pathological detections were snap-frozen in liquid nitrogen and stored at − 80 °C after surgical removal. Samples were obtained from the patients with proper informed consent and approved by the Institutional Review Board of the First Affiliated Hospital of Xiamen University.

### Cell culture and treatment

Human bladder cancer cells lines (RT-112, 5637, BIU-87 and TCCSUP) and human embryonic kidney cells lines (HEK293T) were all purchased from the BNCC cell bank (Beijing, China). Cells were inoculated in Dulbecco’s modified Eagle’s medium (DMEM) supplemented with 10% FBS (GIBCO BRL, NY, USA), 1% Glutamax (35,050, Invitrogen, Carlsbad, CA, USA), 1% Non-essential Amino Acids1 (111,401, Invitrogen) and 1% Sodium Pyruvate 100 mM Solution (113,600,070, Invitrogen). Cultured cells were stored at 37 °C in a humidified atmosphere (5% CO_2_ and 95% O_2_).

### Microarray analysis

Microarray datasets GSE92675 at platform GPL19978 were used. Four pairs of matched bladder tumor tissues and adjacent tissues were prepared for analyzing the expression level and biological functions of circRNAs. Differentially expressed genes were identified using *t*-test (*P* < 0.05) combined with fold change (FC > 2). As for mRNA analysis, 19 pairs of tumor tissues and adjacent tissues obtained from TCGA (https://cancergenome.nih.gov/) were screened by R programme (Fold change> 2, *P* < 0.001).

### MiRNA targets prediction of circCEP128

We predicted the miRNA-binding sites of circCEP128 and *SOX11* using TargetScan (http://www.targetscan.org/) and circular RNA interactome (https://circinteractome.nia.nih.gov/), respectively.

### Cell transfection

TCCSUP and BIU-87 cells were transfected with corresponding plasmids using the Lipofectamine 2000 (Invitrogen) in the light of the manufacturer’s recommendations. And the cells were harvested at 48 h after transfection. Cells were generally assigned to different groups as follows: (1) negative control (NC) group: bladder cancer cells transfected with pCDNA3.1 (GenePharma, Shanghai, China). (2) mimics group: bladder cancer cells transfected with miR-145-5p mimic. (3) inhibitor group: bladder cancer cells transfected with miR-145-5p inhibitor. (4) si-circRNA group: bladder cancer cells transfected with si-circCEP128. (5) si-CEP128 group: bladder cancer cells transfected with si-CEP128. (6) si-*SOX11* group: bladder cancer cells transfected with si-*SOX11*. (7) si-circRNA and inhibitor group: bladder cancer cells transfected with si-circCEP128 and miR-145-5p inhibitor. (8) si-*SOX11* and inhibitor group: bladder cancer cells transfected with si-*SOX11* and miR-145-5p (Thermo Fisher, Waltham, MA, USA). Si-circCEP128 and siCEP128 were designed on Thermo Fisher (https://rnaidesigner.thermofisher.com/) and produced by Sangon Biotech (Shanghai, China) which were shown in Table 1.

**Table 1:** SiRNA sequences

	sense	anti-sense
si-circCEP128	5’-GAGAGCUUGAACAGGAAUU-3’	3’-AAUUCCUGUUCAAGCUCUC-5’
si-CEP128	5’-GCGCTACACCAAATACAAA-3’	3’-UUUGUAUUUGGUGUAGCGC-5’
si-SOX11	5’-GCGAGAAGATCCCGTTCAT-3’	3’-AUGAACGGGAUCUUCUCGC-5’

### qRT-PCR assay

The reverse transcription of RNA was developed using Prime Script RT Master Mix (Takara, Japan). PCR reaction was performed with PCR Master Mix (2×) (ThermoFisher Scientific, Waltham, Ma, USA). For the quantitative determination of circRNA, miRNA and mRNA, real-time PCR analysis was performed using SYBRremix Ex TaqTM kit (Takara, Tokyo, Japan) with GAPDH and U6 as internal controls. All analyses were performed using the StepOnePlus Real-Time PCR System (Applied Biosystems, Carlsbad, CA, USA). The primer sequence was listed in Table 2.

**Table 2:** Primer sequences

Genes	Primers
circCEP128	F: 5’-ACCCACATCGCTGGTTAGC-3’
	R: 5’-TCGATCACCTTCTGCTTTCGT-3’
SOX11	F: 5’-GCCTCTTTTCTGCTGGGTCT-3’
	R: 5’-ACTGAAAACCTCCTCCGCTG-3’
hsa-miR-155-5p	F: 5’-TGCCTCCAACTGACTCCTAC-3’
	R: 5’-GCGAGCACAGAATAATACGAC-3’
hsa-miR-223-3p	F: 5’-GGGGTGTCAGTTTGTCAAA-3’
	R: 5’-CAGTGCGTGTCGCGTGGAGT-3’
hsa-miR-145-5p	F: 5’-CTCACGGTCCAGTTTTCCCA-3’
	R: 5’-ACCTCAAGAACAGTATTTCCAGG-3’
GAPDH	F: 5’-GGAAAGCTGTGGCGTGAT-3’
	R: 5′-AAGGTGGAAGAATGGGAGTT-3’
U6	F: 5’-GCTTCGGCAGCACATATACTAAAAT-3’
	R: 5’-CGCTTCACGAATTTGCGTGTCAT-3’

### Immunohistochemistry

Bladder tissues were immunostained by anti-*SOX11* (1:200, Abcam, Cambridge, MA, USA) and HRP-conjugated goat anti-rabbit IgG (1:1000, Abcam), respectively. The resultant immunostaining images were captured using the AxioVision Rel.4.6 computerized image analysis system (Carl Zeiss, Oberkochen, Germany). Proteins expression levels were analyzed using Image-Pro Plus version 6.0 (Media Cybernetics, MD) by calculating the integrated optical density in each stained area (IOD/area).

### Western blot

Cell lysates were prepared with RIPA buffer (Thermo Scientific). The concentration was determined using a bicinchoninic acid (BCA) protein assay kit (Pierce, Thermo Scientific). Immunoreactive bands were detected by using the Immobilon ECL substrate kit (Millipore, Merck KGaA, Germany). The images were acquired by using BioSpectrum 600 Imaging System (UVP, CA, USA). Antibodies used included primary and secondary antibodies, primary antibodies including anti-*SOX11* (1:1000, Abcam), Bcl-2 (1:500, Abcam), Bax (1:500, Abcam), Cleaved-caspase3 (Anti-active Caspase-3, 1:500, Abcam) and anti-GAPDH (1:10000, Abcam); secondary antibody was HRP-conjugated secondary goat anti-rabbit IgG (1:2000, Abcam).

### Fluorescence in situ hybridization (FISH)

TCCSUP and BIU-87 cells were performed with cytospin, the collected cells were fixed with Carnoy’s fixative (3:1 methanol (ThermoFisher Scientific, Leicestershire, UK): acetic acid (Sigma-Aldrich, Bornem, Belgium) and then air dried for 5 min. CircCEP128 was localized in bladder cells by FISH using CEP Y SpectrumGreen DNA probe (ACCB Biotech, Beijing, China) under the manufacturer’s instructions. After that, the slides with collected cells were mounted with Fluorescence Mounting Medium (Antifade) (Abace Biology, Beijing, China) to counterstain all nucleic on the slide. Subsequently, the slide was scanned at 20-fold magnification using Carl Zeiss Short Distance Plan- Apochromat® objective.

### Flow cytometry (FCM) assay

Transfected cells were subjected to PI staining for detection with Cell Cycle assay Kit (ab112116, Abcam). Then they were subjected to FITC-Annexin V and PI double staining for flow cytometry detection (EPICS, XL-4, Beckman, CA, USA) according to manufacturer’s instructions. Cells were trypsinized, resuspended and incubated with 1.0 μl of PI and 5.0 μl of Annexin V-fluorescein and the apopotosis rate was determined by flow cytometry (FACScan; Becton Dickinson, MountainView, CA, USA) and analyzed with analyzed using Flowjo 7.6 software (BD Bioscience, San Jose, CA, USA).

### MTT assay

Well transfected cells were seeded into 96-well plates at 2 × 10^4^ cells/ml in a 5% CO_2_ atmosphere and incubated overnight. 20 μl MTT reagent (Sigma-Aldrich, Bornem, Belgium) was added to each well respectively at 0, 24, 48 and 72 h. at an absorbance of 490 nm, cell viability was detected with an automatic enzyme-linked immune detector. The experiment was repeated in triplicate.

### Luciferase reporter assay

Dual-luciferase reporter assay system (Promega, Madison, WI, USA) was operated for the co-transfection of HEK293T cells. Mutagens were used to mutate the sequence in 3’UTR of circCEP128 and SOX11. The mutant circCEP128 was transfected with miR-145-5p or empty vector as negative control. The mutant SOX11 was also transfected with miR-145-5p and empty control for two groups. The luciferase results were detected by Luc-Pair™ Duo-Luciferase Assay Kit (Yeasen, Shanghai, China) and the luciferase activity in empty vector cells was normalized as 1.

### RNA pull-down assay

Cells transfected with biotinylated miR-145-5p or mutant miR-145-5p mimics (50 nM) using Lipofectamine RNAiMax (Life Technologies) were harvested and sonicated 48 h after transfection. Remaining cell lysates were incubated with C-1 magnetic beads (Life Technologies) at 4 °C for 2 h and then purified using RNeasy Mini Kit (QIAGEN, Duesseldorf, Germany) for analysis, following detected RNA enrichment by qRT-PCR.

### Tunnel staining

TCCSUP were cultured on coverslips after transfected with miR-145-5p mimics, miR-145-5p inhibitor, si-circRNA, or si-SOX11. 48 h after, cells were harvested and fixed with 4% paraformaldehyde. Cell apoptosis analysis was performed using a tunnel staining kit (Abcam, USA). Cell nuclei were stained with DAPI. All fluorescent images were examined using a Leica DM3000 microscope and photographed using a DFC 420 camera (Leica, Germany).

### EdU incorporation

At the last 18 h of cell culture, Edu-labeling reagent (Invitrogen) (1:1000 dilution) was added into medium. EdU was detected using the Click-iT kit (Invitrogen) following the manufacturer’s protocol. The slides were counterstained with DAPI (4′,6-diamidino-2-phenylindole). Images were captured and analyzed using OpenLab software, and cells were quantitated using ImageJ software.

### Statistical analysis

All data were shown as mean ± standard error of the mean (SEM). Statistical analyses were performed using Graphpad Prism statistical software (Version 6.0; La Jolla, CA, USA). Statistical significance (*P* < 0.05) was determined by Student’s t-test (unpaired) for two-group compared and chi-square test was used to assess the RNA correlation.

## Results

### CircCEP128 (hsa_circ_0102722) and *SOX11* are significantly up-regulated in bladder cancer

**Fig. 1:**
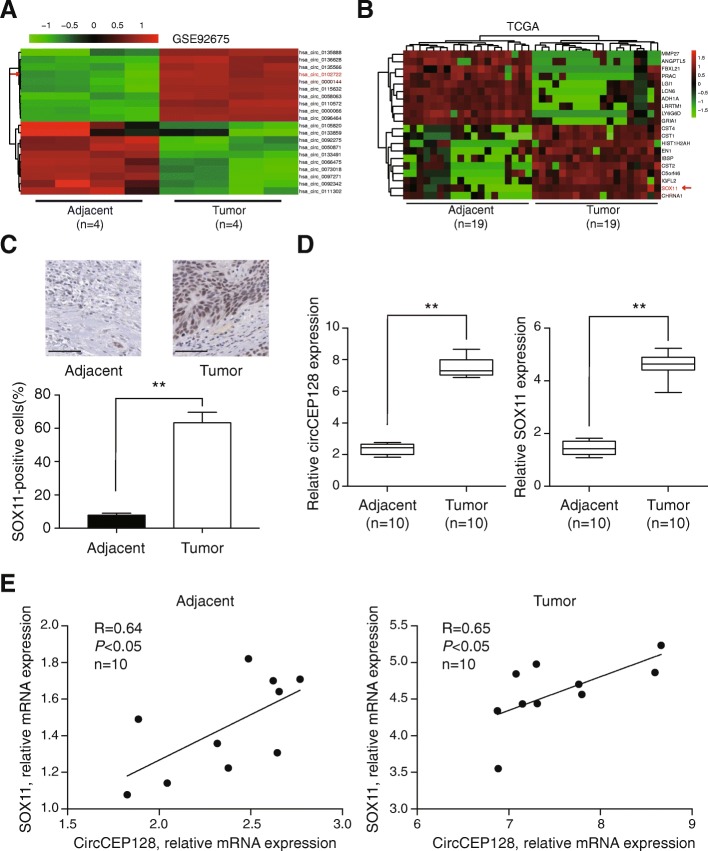
CircCEP128 and *SOX11* are up-regulated in bladder cancer. **a** The cluster heatmap showed some of the differentially expressed circRNAs over 2-fold change between tumor tissues and adjacent normal tissues. Red color indicates high expression level, and green color indicates low expression level. The red arrow indicates hsa_circ_0102722 (circCEP128). **b** The cluster heat map showed some of the differentially expressed mRNAs over 2-old change in tumor tissues and adjacent normal tissues. The red arrow indicates *SOX11*. **c** Immunochemistry staining showed more *SOX11*-positive cells in tumor tissues than in adjacent normal tissues. Scale bar, 50 μm**.** ^**^P < 0.01, compared with adjacent tissues. **d** CircCEP128 and *SOX11* were highly expressed in tumor tissues. ^**^P < 0.01, compared with adjacent tissues. **e** According to the results of qRT-PCR, circCEP128 and *SOX11* were positively related in cancer tissues and in cancer adjacent tissues

A total of 433 circRNAs were differentially expressed (Fold change> 2 and *P* < 0.05) with the analysis of circRNA microarray from GEO database. 169 circRNAs were significantly down-regulated, and 264 circRNAs were up-regulated in 4 bladder cancer samples. The top ten up- and down-regulated circRNAs were chosen by fold change to draw the cluster heat map (Fig. 1a). CircCEP128 was up-regulated in bladder tumor tissues with a fold change value of 5.06 (Table 3). Data of 19 pairs sample (tumor and adjacent tissue of patients) were obtained from TCGA and analyzed by R software, the cluster heat map was drawn (Fig. 1b). *SOX11* was up-regulated in bladder tumor tissues with a fold change value of 11.56 (Table 4). Immunochemistry staining showed more *SOX11*-positive cells in tumor tissues than that of adjacent normal tissues (Fig. 1c, *P* < 0.01). The highly expressions of circCEP128 and SOX11 were validated in 10 paired of bladder tissues by RT-PCR (Fig. 1d, *P* < 0.01). Analysis of clinicopathologic features, circCEP128 and *SOX11* showed significant higher expressions in bladder cancer tissues compared with adjacent normal tissue (Table 5). Gender and age had no relationships with the expression of circCEP128 or *SOX11*except for tumor size, TNM stage and lymphatic metastasis. CircCEP128 and *SOX11* had a positively related dependency both in cancer tissues and in adjacent normal tissues (Fig. 1e, *P* < 0.05). Thus we hypothesized that circCEP128 may function as a ceRNA (competing endogenous RNAs) and promote the expression of *SOX11*.

**Table 3:** Log_2_ (Fold Change) of ten increased circular RNA and ten decreased circular RNA

Circular RNA	Log_2_(Fold Change)
hsa_circ_0135888	4.88
hsa_circ_0136628	4.93
hsa_circ_0135566	4.97
hsa_circ_0102722	5.06
hsa_circ_0000144	5.71
hsa_circ_0115632	4.64
hsa_circ_0058063	4.85
has_circ_0110572	5.30
hsa_circ_0000066	5.62
hsa_circ_0096464	5.30
hsa_circ_0105820	− 3.03
hsa_circ_0133859	−2.63
hsa_circ_0092275	−2.71
hsa_circ_0050871	−2.63
hsa_circ_0133491	−2.89
hsa_circ_0066475	−4.10
hsa_circ_0073018	−3.41
hsa_circ_0097271	−3.44
hsa_circ_0092342	−3.83
hsa_circ_0111302	−3.24

The positive number means circular RNA was increased in tumor samples, the minus means circular RNA was decreased in tumor samples

**Table 4:** Log_2_ (Fold Change) of ten increased mRNA and ten decreased mRNA

mRNA	Log_2_ (Fold Change)
MMP27	−14.42
ANGPTL5	−12.50
FBXL21	−13.47
PRAC	−14.15
LGI1	−12.83
LCN6	−13.61
ADH1A	−12.92
LRRTM1	−13.19
LY6G6D	−14.23
GRIA1	−12.39
CST4	10.29
CST1	10.75
HIST1H2AH	10.68
EN1	10.96
IBSP	11.36
CST2	12.33
C5orf46	13.47
IGFL2	11.94
SOX11	11.56
CHRNA1	10.61

The positive number means mRNA was increased in tumor samples, the minus means mRNA was decreased in tumor samples

**Table 5:** The relationship between circCEP128/SOX11 expression and clinicopathological characteristics in bladder cancer patients

Items	Patients (*N* = 10)	circCEP128 RNA (X ± SD)	*P* value	SOX11 mRNA (X ± SD)	*P* value
N	10	0.00563 ± 0.00235	0.0038^b^	3.2565 ± 2.0583	0.0032^b^
T	10	0.02568 ± 0.01897		6.0354 ± 1.5568	
Gender			0.7704		0.4643
Male	4	0.02258 ± 0.01989		3.3564 ± 3.0254	
Female	6	0.01897 ± 0.01765		4.8697 ± 3.0658	
Age (years)			0.3785		0.463
< 60	2	0.00897 ± 0.00458		2.7856 ± 1.8975	
> =60	8	0.02305 ± 0.02035		5.6578 ± 4.9876	
Tumor size			0.0291^a^		0.0168^a^
< 10	5	0.01756 ± 0.00968		1.8756 ± 0.9873	
> =10	5	0.03457 ± 0.01057		5.5893 ± 4.5760	
TNM stage			0.0361^a^		0.0263^a^
I-II	3	0.01687 ± 0.00952		3.8976 ± 1.0687	
III-IV	7	0.04356 ± 0.01689		6.3654 ± 1.3873	
Lymphatic metastasis			0.0313^a^		0.0343^a^
NO	3	0.00897 ± 0.00563		1.7056 ± 0.9856	
Yes	7	0.03056 ± 0.01347		6.0325 ± 2.7846	

*N*: Adjacent normal tissues; *T*: Bladder cancer tissues; Student-*t* test

^a^*P* < 0.05, ^b^*P* < 0.01 was recognized as a significant difference

### CeRNA analysis for circCEP128 according to the database

**Fig. 2:**
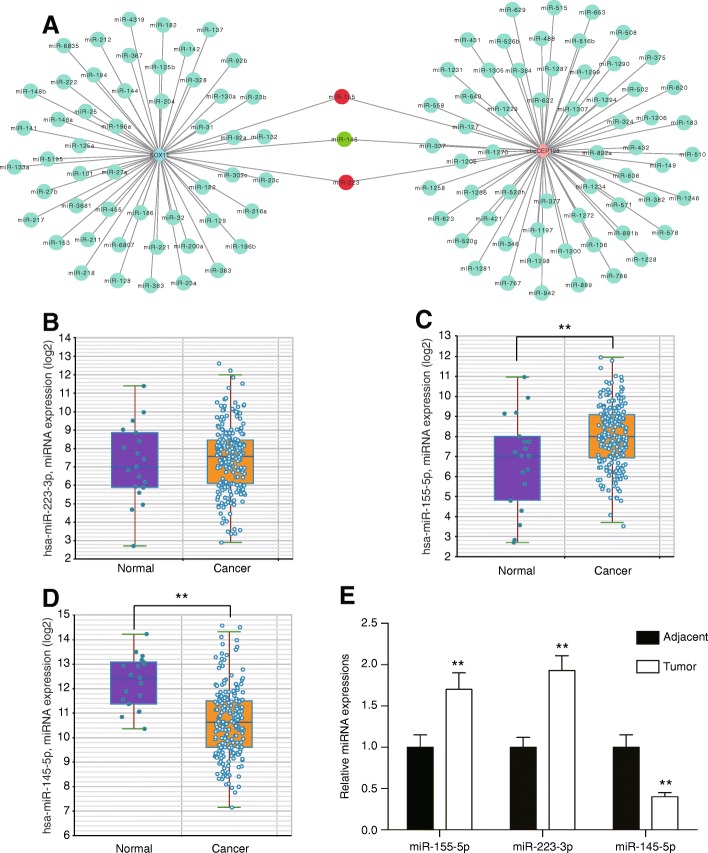
CeRNA analysis for circCEP128. **a** Cytoscope was used to visualize circCEP128-miRNA-target gene interactions based on the TargetScan and circular RNA interactome. Red color indicates high expression level in bladder cancer and green color indicates low expression level in bladder cancer. **b-d** TCGA bladder cancer patients’ data analysis was that miR-223-3p expression had no significant difference, miR-155-5p expression increased but miR-145-5p expression decreased in tumor samples. ^**^P < 0.01, compared with adjacent samples. **e** QRT-PCR results showed that miR-155 and miR-223 was up-regulated in tumor tissues while miR-145 was down-regulated in tumor tissues. ^**^P < 0.01, compared with adjacent tissues

In Fig. 2a, miR-155, miR-223 and miR-145 were found to have the same targeting relations with circCEP128 and *SOX11* by using circular RNA interactome (https://circinteractome.nia.nih.gov/Circular_RNA/circular_rna.html) and TargetScan (http://www.targetscan.org/). Firstly, the analysis results had been verified in database of starBase v2.0 (http://starbase.sysu.edu.cn/) with TCGA data in bladder cancers, which showed that miR-155-5p obviously up-regulated while miR-145-5p obviously down-regulated in tumor cells (*P* < 0.01, Fig. 2c and d), miR-223-3p expression had no statistically change between normal and tumor cells (*P >* 0.05, Fig. 2b). Subsequently, the experiments of qRT-PCR were conducted in 10 bladder cancer tissues (Fig. 2e). It showed that miR-155-5p, miR-233-3p were upregulated and miR-145-5p was downregulated in tumor cells (*P* < 0.01). The down-regulation of miR-145-5p was speculated as a competitive target of circCEP128 and *SOX11* in bladder cancer.

### CircCEP128 serves as a sponge of miR-145-5p which directly regulates *SOX11*

**Fig. 3:**
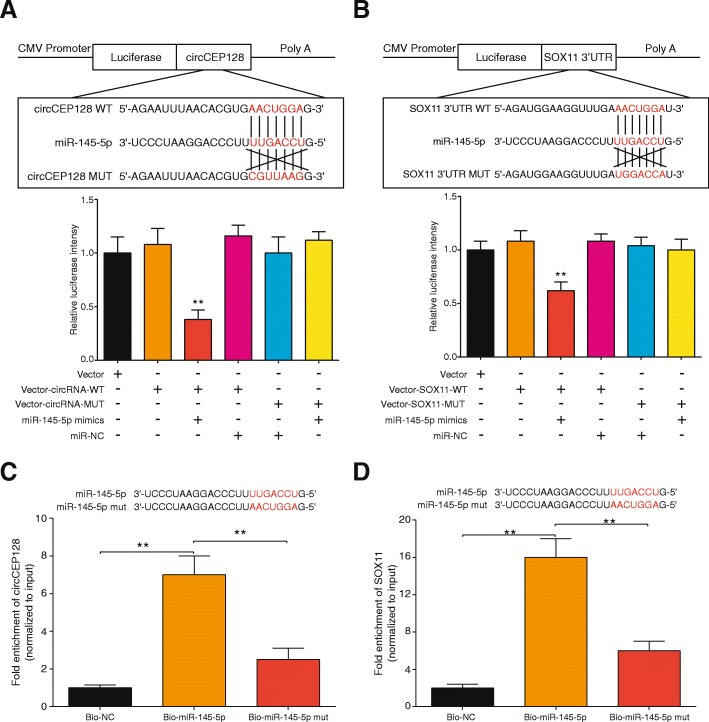
CircCEP128 serves as a sponge of miR-145-5p and indirectly targets at *SOX11*. **a** and **b** Binding sites of miR-145-5p in circCEP128/SOX11 3’UTR (Up). Luciferase reporter assay revealed that miR-145-5p was able to reduce the luciferase intensity more than 40% by targeting at wild type RNAs (WT, Down). ^**^P < 0.01, compared with the group with only vector. **c** and **d** RNA pull-down assays indicated the physical interactions between circCEP128 and miR-145-5p or between miR-145-5p and *SOX11*

StarBase v2.0 predicted the binding relationship between CircCEP128 and miR-145-5p. Luciferase reporter assay showed that the luciferase intensity of HEK293T cells transfected with circCEP128 wild type or miR-145-5p mimics were significantly reduced, but the luciferase intensity of cells transfected with circCEP128 muted type or miR-145-5p mimics were hardly changed (*P* > 0.05, Fig. 3a). Similarly, with the targetScan prediction of the binding sequences of miR-145-5p and *SOX11*, it showed that the luciferase activity was significantly reduced in *SOX11* wild type + miR-145-5p mimics co-transfection group (*P* < 0.01, Fig. 3b). After RNA pull-down assay, the circCEP128 level of the miR-145-5p group was 6.73 times as much as NC group and 2.28 times as much as miR-145-5p-mut group (*P* < 0.01, Fig. 3c) and the miR-145-5p group was found high *SOX11* level which was 16.69 times as much as NC group (*P* < 0.01, Fig. 3d).

### Knockdown of circCEP128 inhibits expression of *SOX11*

**Fig. 4:**
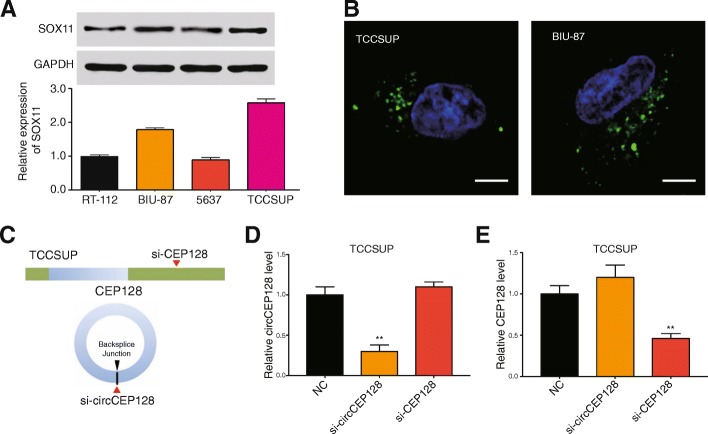
Si-circCEP128 inhibits the expression of circCEP128 not CEP128. **a** SOX11detection of 4 bladder cancer cells was conducted by qRT-PCR. **b** Cellar locations of circCEP128 were in TCCUSP and BIU-87 cytoplasm. Scale bar, 2 μm**. c** Schematic model of the si-circRNAs. si-CEP128 targets the CEP128 linear transcript, si-circCEP128 targets the back-splice junction of circCEP128. **d** and **e** Si-circCEP128 knocked down only the circular transcript and did not affect the expression of linear species. Si-CEP128 knocked down only the CEP128 linear transcript but not the circular transcript. ^**^P < 0.01, compared with NC

**Fig. 5:**
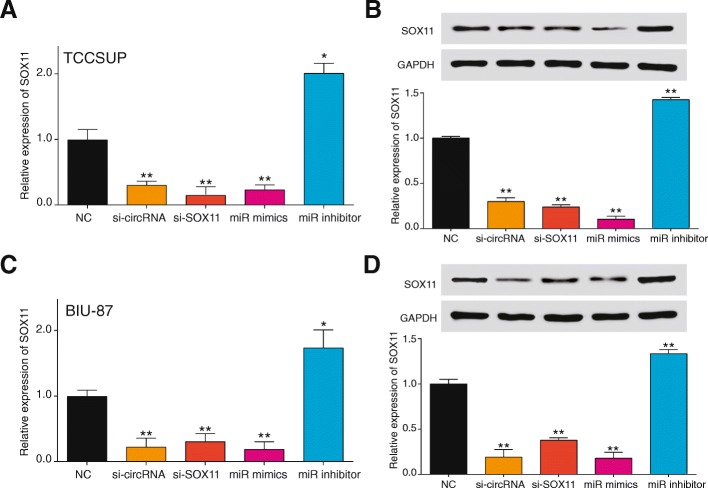
Knockdown of circCEP128 inhibits expression of *SOX11*. The expression levels of *SOX11* were detected following knockdown of circCEP127 using si-circCEP128, miR-145-5p inhibitor, co-transfection with si-circCEP128 and the miR-145-5p inhibitor or si-*SOX11* (**a**) in TCCSUP and (**c**) in BIU-87. ^**^P < 0.01, compared with NC. *SOX11* protein relative expression levels were analyzed by western blotting (**b**) in TCCSUP and (**d**) in BIU-87. ^**^P < 0.01, compared with NC

In bladder cancer cell lines, SOX11 expressed higher in BIU-87 and TCCSUP compared with RT-112 and 5637 (Fig. 4a). TSSCP showed the highest expression level of SOX11. Experiments of fluorescent probe localized circCEP128 in the plasma of bladder cell lines (Fig. 4b). Si-circCEP128 (Fig. 4c) could knockdown the expression of circCEP128 while si-CEP128 had no effect on altering the expression of circCEP128 (*P* > 0.05, Fig. 4d). Similarly, Si-circCEP128 had no effect on regulating the expression of CEP128, while si-CEP128 could knockdown the expression of CEP128 (*P* < 0.01, Fig. 4e). Expression of *SOX11* was detected by qRT-PCR (*P* < 0.01, Fig. 5a and c) and western blot (*P* < 0.01, Fig. 5b and d) in TCCSUP and BIU-87 cells. Expression of SOX11 was increased greatly in miRNA inhibitor group while decreased dramatically in si-SOX11 group. The results verified that knockdown of circCEP128 inhibited expression of *SOX11* and it could be guessed that knockdown of circCEP128 promotes expression of miR-145-5p to inhibit expression of *SOX11*.

### The effect of circCEP128 on bladder tumor cell proliferation, apoptosis and cell cycle

**Fig. 6:**
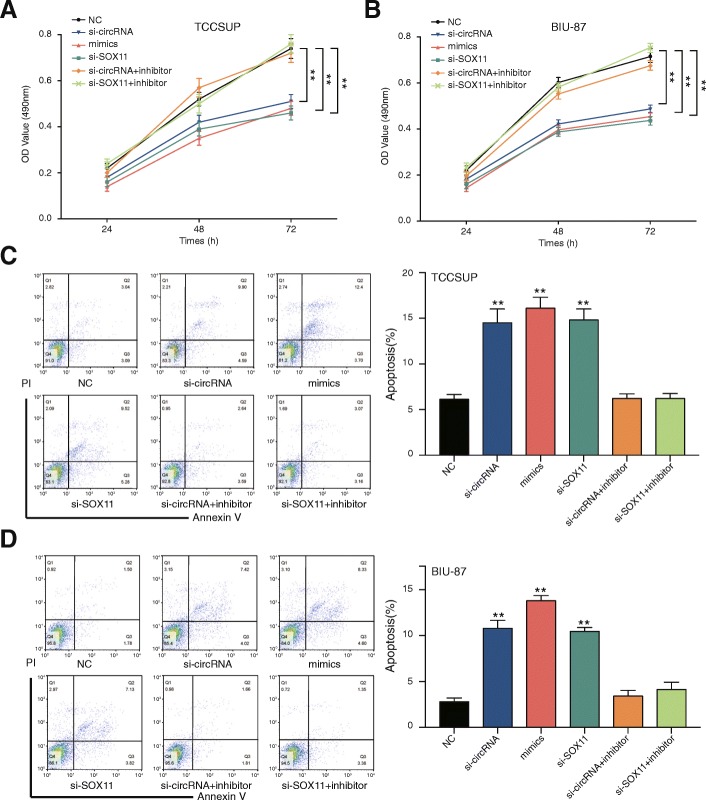
Effects of circCEP128 on cell proliferation. MTT assay was also performed to assess cell proliferation after transfection with si-circRNA of circCEP128, miR-145-5p mimics, si-*SOX11*, co-transfection with si-circRNA and the miR-145-5p inhibitor and co-transfection with si-*SOX11* and the miR-145-5p inhibitor (**a**) in TCCSUP and (**b**) in BIU-87. Knockdown of circCEP128 inhibited cell proliferation. ^*^P < 0.05, compared with NC. Cell apoptosis was analyzed using flow cytometry (**c**) in TCCSUP and (**d**) in BIU-87 with double staining of PI and Annexin V-FITC. Knockdown of circCEP128 induced the increased cell apoptosis rate. ^**^P < 0.05, compared with NC

**Fig. 7:**
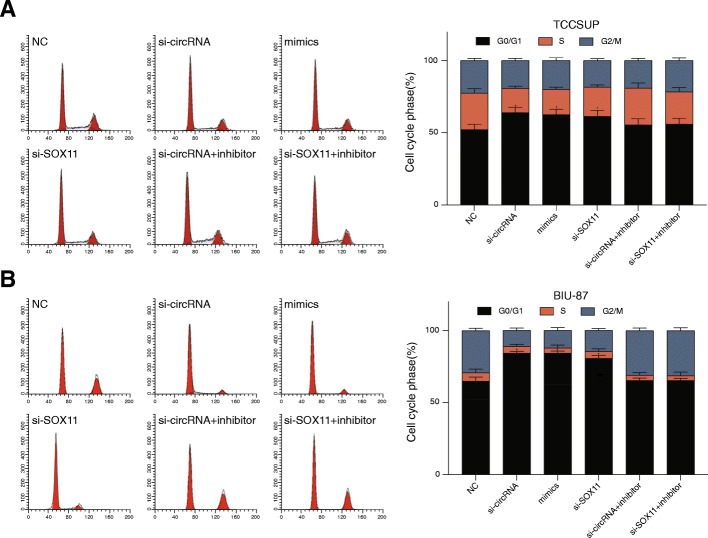
Effects of circCEP128 on cell cycle. Cell cycle was analyzed using flow cytometry after transfection with si-circRNA of circCEP128, miR-145-5p mimics, si-*SOX11*, co-transfection with si-circRNA and the miR-145-5p inhibitor and co-transfection with si-*SOX11* and the miR-145-5p inhibitor (**a**) in TCCSUP and (**b**) in BIU-87. Knockdown of circCEP128 induced G1/S arrest. ^*^P < 0.05, compared with NC

Knockdown of circCEP128 inhibited cell proliferation (*P* < 0.05, Fig. 6a and b) but promoted cell apoptosis (*P* < 0.05, Fig. 6c and d). Cell cycle induced bladder cancer cell G1/S arrest in si-cirRNA, miR-145-5p mimics and si-*SOX11* group (*P* < 0.01, Fig. 7a and b). Similar results were generated after improving expression of miR-145-5p or knocking down *SOX11* (*P* < 0.01, Fig. 6a-Fig. 7b). Cell cycle, cell apoptosis rate and cell proliferation were recovered with the addition of miR-145-5p inhibitor after knocking down the circCEP128 (*P* > 0.05, Fig. 6a-Fig. 7b). Similarly, Cell cycle, cell apoptosis rate and cell proliferation were recovered with si-*SOX11* after the inhibition of miR-145-5p (*P* > 0.05, Fig. 6a-Fig. 7b). As a result, knockdown of circCEP128 induced the inhibition of cell proliferation and increased bladder cancer cell apoptosis rate.

### Knockdown of circCEP128 promoted TCCSUP cells damage and apoptosis

**Fig. 8:**
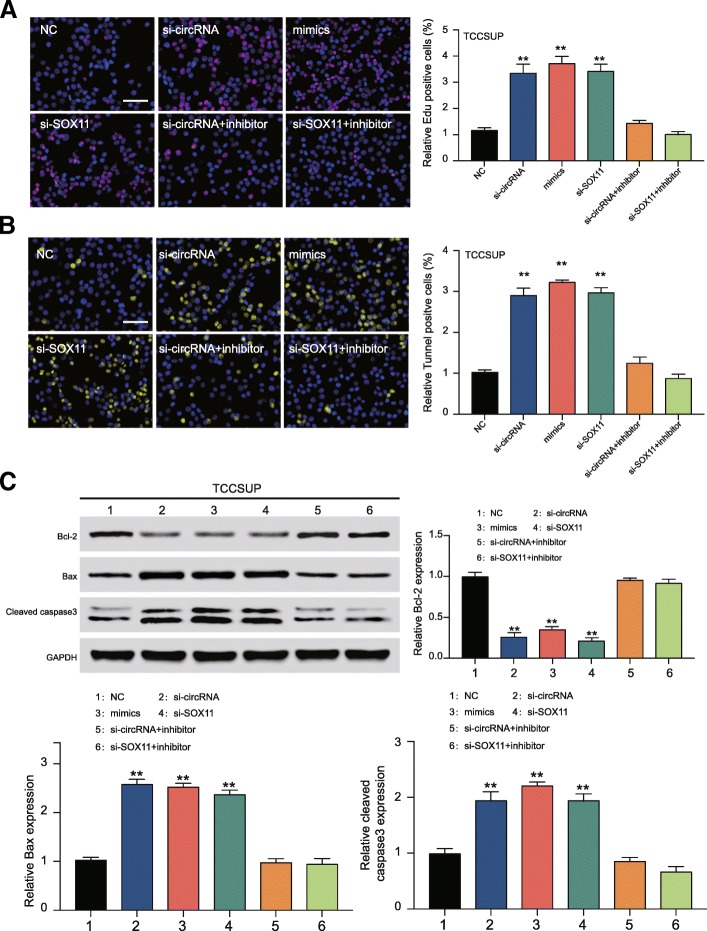
Effects of circCEP128 on TCCSUP cell damage and apoptosis. After transfected with corresponding siRNA or mimics/inhibitor, higher DNA damage happened in si-circRNA, miR-145-5p mimics and si-*SOX11* with the experiments of **a** Edu staining **b** Tunnel staining. ^**^P < 0.01, compared with NC. Scale bar, 20 μm. c Western blot showed the result of apoptosis related proteins bax and caspase3 were higher but bcl-2 was lower in si-circRNA, miR-145-5p mimics and si-*SOX11* groups compared with NC group. ^**^P < 0.01, compared with NC

Damaged TCCSUP cells marked Edu and Tunnel staining, a strong positive cells presented in si-circRNA, miR-145-5p mimics and si-*SOX11* group (*P* < 0.01, Fig. 8a and b). The effects of si-circRNA and si-SOX11 on TCCSUP had been recovered by miR-145-5p inhibitor. The expression levels of three apoptosis relative proteins were detected (Fig. 8c), the results showed that the expression of Bcl-2 was significantly decreased in si-circRNA group, mimics group as well as si-SOX11 group (*P* < 0.01). However, the expression levels of bax and cleaved caspase3 were increased obviously in si-circRNA group, mimics group and si-SOX11 group (*P* < 0.01).All in all, inhibition of circCEP128 induced TCCSUP cell damage and apoptosis through the regulation of miR-145-5p/SOX11.

## Discussion

In this study, circCEP128 and *SOX11* were found highly expression and positively related in bladder cancer tissues. The relation could be regulated by miR-145-5p, a downregulated miRNA in bladder cancer samples. CircCEP128 served as a sponge of miR-145-5p and indirectly regulated *SOX11*. Knockdown of circCEP128 could induce the inhibition of cell proliferation and increase bladder cancer cell apoptosis rate.

Many studies have confirmed that stable transcripts with a host of miRNA-binding sites or miRNA response elements (MREs) could function as miRNA sponges (Memczak et al., 2013) and circular RNAs are found to be enriched in functional miRNA binding sites (Huang et al., 2016). For example, Li et al. found that circHIPK3 inhibited migration, invasion and angiogenesis of human invasive bladder cancer by targeting miR-558 (Markopoulos et al., 2017). In addition, other studies indicated that circRNA-MYLK could function as an endogenous sponge for miR-29a in bladder cancer (Zhong et al., 2017). What is more, circRNA CDR1as was reported to negatively regulate miR-7 and increase the levels of miR-7 targets (Xu et al., 2015; Hansen et al., 2013). These conclusions coincide with the results of our study, which indicate that circCEP128 is able to function as a ceRNA for miR-145-5p.

Recently, studies about circular RNA mainly focus on its interactions with miRNA and miRNA targets. For example, it is revealed that circRNA-MYLK functions as a ceRNA for miR-29a, thus boosting *VEGF/VEGFR2* expressions and activating downstream Ras/ERK signaling pathway in bladder cancer progression (Zhong et al., 2017). Li et al. demonstrated that cir-ITCH increased the expression of the miRNA target gene *ITCH* in esophageal squamous cell carcinoma (Li et al., 2015). Other studies showed that the over-expression of circHIPK3 induced efficiently interaction with miR-558 and then down-regulated the expression of *HPSE* and its downstream targeted *MMP-9* and *VEGF* to attenuate the promoting effect of miR-558 on bladder cancer cell migration, invasion, and angiogenesis (Li et al., 2017). Also, it was speculated that circTCF25 may competitively bind with miR-103a-3p and miR-107 and relieve their suppression on associated target genes (Zhong et al., 2016). Similarly, in our study, we found that circCEP128 served as a sponge of miR-145-5p and indirectly regulated *SOX11*.

*SOX11* have been proved to promote invasive growth and progression of DCIS cells (Oliemuller et al., 2017) and prevent cell differentiation in mantle cell lymphoma (Meggendorfer et al., 2013). However, Sernbo et al. demonstrated that the overexpression of *SOX11* could induce growth arrest in ovarian cancer cells (Sernbo et al., 2011). Varied findings may result from differential populations and cancer types (Fang et al., 2017). In this study, *SOX11* was a contributor to bladder cancer in terms of proliferation and apoptosis. Therefore, it was verified that circCEP128 acted as a ceRNA for miR-145-5p to regulate *SOX11*, which further promoted cell proliferation and suppressed cell apoptosis of bladder cancer.

In this study, siRNA could only specifically target the circular form but not the linear form of CEP128; thus the off-target effect have to be considered. Therefore, more effective, accurate and specific methods of RNA interference remain to be exploited. Additionally, further research on functions and mechanisms underlying circCEP128 are required.

## Conclusion

In conclusion, the results of our study demonstrate that circCEP128 is up-regulated in human bladder cancer, and is able to sponge miR-145-5p for promoting *SOX11* expression with high efficiency. We also demonstrate that knockdown of circCEP128 can effectively inhibit cell proliferation and promote cell apoptosis rate of bladder cancer cells through targeting miR-145-5p/*SOX11* axis. Our findings provide novel evidences that circRNAs might act as “microRNA sponges” and provide a new therapeutic target for the treatment of bladder cancer.

## Abbreviations

BCA: Bicinchoninic acid
ceRNA: Competing endogenous RNA
circCEP128: Circular RNA CEP128
circRNAs: Circular RNAs
FCM: Flow cytometry
FISH: Fluorescence in situ hybridization
miR-145: MicroRNAs
miRNAs: MicroRNAs
MREs: MiRNA response elements
NC: Negative control
SEM: Standard error of the mean
